# A Multiaxial Fracture of Ecoflex Skin with Different Shore Hardness for Morphing Wing Application

**DOI:** 10.3390/polym15061526

**Published:** 2023-03-20

**Authors:** Dilshad Ahmad, Rafic M. Ajaj

**Affiliations:** 1 Department of Aerospace Engineering, Khalifa University of Science and Technology, Abu Dhabi 127788, United Arab Emirates; 2 Advanced Research and Innovation Center (ARIC), Department of Aerospace Engineering, Khalifa University of Science and Technology, Abu Dhabi 127788, United Arab Emirates

**Keywords:** ecoflex, morphing skin, crack propagation, morphing wing, fracture

## Abstract

The use of elastomer-based skins in morphing wings has become increasingly popular due to their remarkable stretchability and mechanical properties. However, the possibility of the skin fracturing during multiaxial stretching remains a significant design challenge. The propagation of cracks originating from flaws or notches in the skin can lead to the specimen breaking into two parts. This paper presents an experimental study aimed at comprehensively evaluating crack propagation direction, stretchability, and fracture toughness of silicone-based elastomeric skin (Ecoflex) for morphing wing applications, using varying Shore hardness values (10, 30, and 50). The findings show that the lower Shore hardness value of 10 exhibits a unique Sideways crack propagation characteristic, which is ideal for morphing skins due to its high stretchability, low actuation load, and high fracture toughness. The study also reveals that the Ecoflex 10 is suitable for use in span morphing, with a fracture toughness of approximately 1.1 kJ/m${}^{2}$ for all thicknesses at a slower strain rate of 0.4 mm/min. Overall, this work highlights the superior properties of Ecoflex 10 and its potential use as a morphing skin material, offering a groundbreaking solution to the challenges faced in this field.

## 1. Introduction

With the exponential growth of the aviation industry in recent times, it is imperative to curtail greenhouse gas emissions [1,2]. As a result, novel technologies are being implemented, including lightweight structures, improved aerodynamics, and enhanced propulsion efficiency, to minimize fuel usage [3,4,5]. Among these technologies, the lightweight morphing wing holds great promise. It dynamically adjusts its shape to reduce drag and increase lift generation, but its most challenging aspect is its flexible skin, which is wrapped over ribs and structures, enabling conformal and seamless shape change [6]. The flexible skin provides significant benefits, such as reducing complex structure, providing seamless and noise-free operation, and incurring minimal frictional losses. Consequently, numerous researchers have explored the use of elastomer-based skins to develop morphing skin, highlighting its tremendous potential.

The field of morphing wings owes its success to the pioneering work of Kikuta [7], who conducted an extensive range of mechanical tests on various materials, including polyurethane (Tecoflex 80A), to demonstrate its efficacy in morphing wings. Thill et al. [8] went on to review multiple materials suited to morphing skins and concluded that elastomer-based skins could be a potential design solution as they offer both flexibility and stiffness. Subsequently, Bubert et al. [9] successfully implemented a silicone-based elastomer skin for a span morphing wing, achieving a remarkable 100% increase in span for a 100% increase in surface area with a negligible poisson’s ratio. The outstanding properties of elastomers continued to attract researchers’ attention, with Kuduva [10] utilizing silicone elastomers in the morphing wing to achieve a high actuation rate, less actuation force, and multiaxial shape change with extreme stretchability. Olympio and Gandhi [11,12,13] combined elastomers with various core designs to achieve higher global strains than those of the base material. Additionally, extensive experimental and theoretical studies on elastomer-based skins were conducted by Ajaj et al. [14,15,16], proposing the Zigzag wingbox concept and Active Span morphing and Passive Pitching (ASAPP), respectively, using elastomeric skins wrapped over the ribs and structures of the morphing wing.

The literature studies mentioned above strongly indicate that the use of elastomer-based skins in the morphing wing is highly advantageous. Elastomers offer exceptional properties such as high out-of-plane stiffness to sustain aerodynamic loads, low in-plane stiffness for minimal actuation force, extreme stretchability, as well as being lightweight with high fracture toughness, allowing for exceptional manoeuvrability of the morphing wing. Nevertheless, elastomers can be susceptible to nonlinear viscoelastic deformation and fracture in the presence of flaws and notches. Thus, numerous researchers have conducted extensive investigations to characterize the elastomers under different conditions. In particular, Ahmad and Ajaj [17] established that pure shear mode of deformation generates the least hysteresis losses, while Ahmad et al. [6] recommended the silicone-based Ecoflex elastomer as the ideal morphing skin due to its low hysteresis losses and actuation force compared to other elastomers. Furthermore, Liao [18] demonstrated that the stiffness, hysteresis, relaxation time, and equilibrium stress of Ecoflex elastomer increase with increasing Shore hardness. Liao et al. [19] also investigated the stress softening and recovery behavior of Ecoflex elastomer under different deformation modes and Shore hardness. Additionally, Ahmad et al. [20] studied the flaw sensitivity of Ecoflex-30 and VHB under pure shear loading and showed that Ecoflex-30 has higher stretchability, fracture toughness, and failure stress than VHB within the flaw-sensitive length. Ahmad et al. [21] also investigated the fracture properties of Ecoflex-30 and VHB under biaxial loading and found that the fracture toughness and stretchability under biaxial loading decrease with an increase in draw ratios. Recently, Lee and Pharr [22] conducted fracture testing on Ecoflex-30 and concluded that the sideways crack occurs in thick specimens at lower strain rates.

The literature currently lacks comprehensive analysis of the fracture behavior of silicone-based elastomers (Ecoflex) in the context of morphing skin, despite its widespread use in various applications. The risk of fracture during stretching in the morphing wing necessitates careful consideration of crack propagation direction to ensure safety and stretchability. Recognizing this gap in the literature, the current study represents a pioneering effort to thoroughly investigate the fracture toughness and stretchability of Ecoflex for various Shore hardness values under pure shear loading in the context of morphing wings. Additionally, this study is the first to successfully explore the occurrence of sideways crack propagation, which is crucial for preventing failure in multiaxial fracture modes and other relevant parameters. To achieve this, standard force-displacement curves were generated for varying Shore hardness values with a fixed notch at different strain rates and thicknesses. Moreover, stretchability, actuation force, and crack propagation direction were investigated for the morphing skin application. In this regard, the current study is structured as follows: Section 2 provides a detailed discussion of the experimental methodology, including the synthesis of materials, fixture details, and setup methodology, and elaborates on forward and sideways crack propagation for different Shore hardness values and strain rates under multiaxial deformation modes. The results of the study, which include a comprehensive analysis of fracture toughness for various strain rates and Shore hardness values in the form of a table, are presented in Section 3. Moreover, Section 4 outlines potential avenues for future research, while Section 5 summarizes the findings of this study in the context of morphing wings.

## 2. Experimental

### 2.1. Material Synthesis, Fixture and Set Up Details

The materials used for the testing are Ecoflex 10, Ecoflex 30, and Ecoflex 50. The numerical prefixes decide the Shore hardness of the Ecoflex. These are platinum-catalyzed silicones that can be cured at room temperature. During the synthesis of the material, we follow the optimum condition specified by the manufacturer (Smooth on) on their website (www.smooth-on.com, accessed on 19 December 2022). The synthesis of Ecoflex elastomer with different Shore hardness is made in a mold of 10 mm thick acrylic sheet. The mold is fabricated in the acrylic sheet by creating pockets of 0.61 mm, 2.1 mm and 3.61 mm. The extra 1 mm is kept to compensate the shrinkage when the material solidifies. To synthesize the Ecoflex sheets, two parts (Part A and B) of Ecoflex are mixed together and stirred continuously for 3–4 min. After proper mixing, the mixture is uniformly poured into each mold. The poured mixture is then evenly distributed with the help of straight plates so that excess mixture falls down the plate to prevent the formation of any trapped bubbles. The mixture is then allowed to cool at room temperature for about 4 h in each case. In this way, Ecoflex sheets with different thicknesses and varying Shore hardness are prepared. The thickness of each sheet is then measured with a portable thickness micrometer (Model: Yunir1z5xbr97ut) from different parts of the sheets. The variation of thickness is found to be in the range of 0.6 mm ± 0.05 μm, 2 mm ± 0.04 μm, and 3.6 mm ± 0.04 μm. The Ecoflex sheet is then ready to test in the UTM machine.

**Figure 1:**
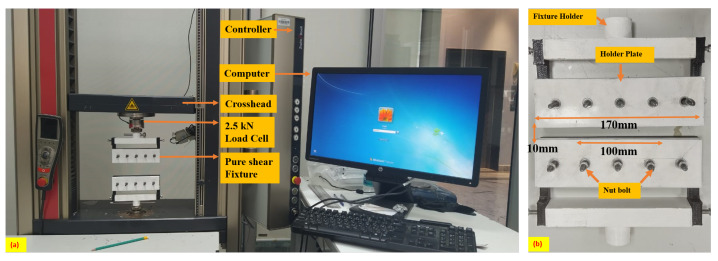
Details of (**a**) UTM (Zwick Roell) and experimental setup used for pure shear and uniaxial fracture experiments. (**b**) Enlarged view of laboratory made 3D printed fixture used for fracture experiment.

Two especially pure shear fixtures (PLA material) are printed in a 3D printer (Model: v3, Make: BigRep ONE, Berlin, Germany) to be utilized in the universal testing machine (UTM) as shown in Figure 1a. The width of the fixture is 170 mm. The pure shear specimen used in the fixture has a dimension of 100 mm width and 10 mm height to maintain the width-to-height ratio equal to 10. The crack length is kept at 20% of width, i.e., 20 mm. A 100 mm wide sandpaper is fixed in the fixture to provide proper gripping to the sample as shown. The fixture is fixed in the UTM through fixture holders located at the top attached to the 2.5 kN load cell as shown in Figure 1a. The specimen is placed between two plates (black and white color) of the fixture as shown in Figure 1b. Furthermore, both plates are tightened keeping the specimen sandwiched between them with five nuts and bolts in each fixture. After tightening the nut and bolts, the fixtures are fixed in the UTM (Model: Z005, Make: Zwick/Roell, Ulm, Germany). The UTM has a load cell of capacity 2.5 kN. After fixing the fixtures in the upper and lower grippers, the grip-to-grip separation is kept at 10 mm. The value of strain rate, thickness, and width of the sample is fed into the TestXpert software (Version: 3.2). integrated with the UTM through a controller as shown in Figure 1a. After accepting the grip-to-grip separation, the force value is made to zero in the software, and then the experiment is started. The crosshead of the UTM moves the upper fixture in the vertical direction keeping the lower fixture fixed. This enables the tearing experiment of specimens in the UTM as shown in Figure 1a. Each test is repeated at least three times to confirm reproduciblity, accuracy and validity of the data obtained.

### 2.2. Forward Crack Propagation in Pure Shear Loading

**Figure 2:**
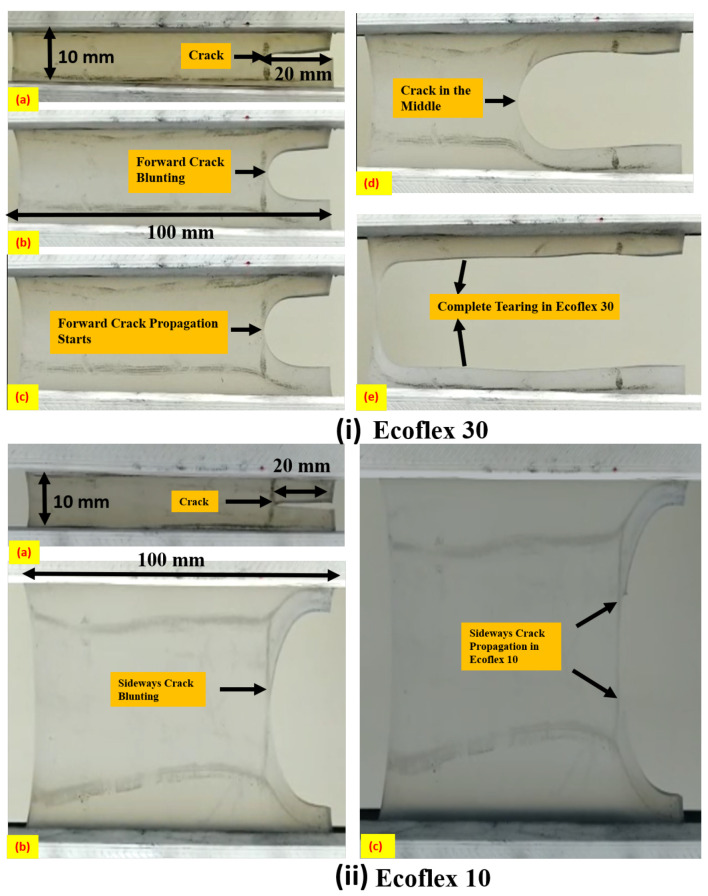
Representation of (**i**) forward crack propagation of Ecoflex 30 at a strain rate of 40 mm/min; (**a**) 20 mm notch length; (**b**) blunting of forward crack; (**c**) starting of forward crack propagation; (**d**) crack reaches at the middle of the specimen; (**e**) tearing of Ecoflex 30 in two parts. (**ii**) Sideways crack propagation of Ecoflex 10 of thickness 0.6 mm at a strain rate of 40 mm/min; (**a**) 20 mm initial notch; (**b**) blunting of sideways crack; (**c**) propagation of sideways crack.

Figure 2i demonstrates an instance of forward crack propagation in Ecoflex 30, under pure shear loading at a fixed strain rate of 40 mm/min. The creation of a notch in the specimen is depicted in Figure 2(ia), and subsequently, the crack begins to blunt, as demonstrated in Figure 2(ib). The chains and entanglements of the Ecoflex are fully stretched, following which chain initiation takes place, as shown in Figure 2(ic). The crack propagates in a blunted form in the direction of the notch, as illustrated in Figure 2(id), ultimately causing the specimen to rupture into two parts. This type of crack propagation, where the crack advances in the direction of the notch, is commonly referred to as ’forward crack propagation.’ It is noteworthy that in this type of crack propagation, the stretchability of the specimen with a crack is relatively less.

### 2.3. Sideways Crack Propagation in Pure Shear Loading

The behavior of crack propagation and its rate in Ecoflex elastomers is distinct from that of other solid materials such as fiber-reinforced polymers [23,24,25,26,27,28] and other materials [29,30]. In most cases mentioned earlier, crack initiation begins from the designated notch position and travels parallel to it. The same behavior is observed for Shore hardness 30 and 50, as shown in Section 2.2 Moreover, the rate of crack propagation in elastomers such as Ecoflex is very high due to its extreme stretching before catastrophic failure. It is worth mentioning that during the experiment, the rate of forward crack propagation was higher than that of sideways crack propagation. However, a comprehensive comparative analysis of crack propagation rates is beyond the scope of this study. The viscoelastic nature and chain alignment of Ecoflex cause strain hardening, resulting in its extreme stretching. Furthermore, due to stretching, there is a strong possibility of faster chain alignment and significant strain hardening in Shore hardness 10, which resists crack growth in the forward direction. As a result, the breaking of chains and entanglements per unit length of propagation is higher [22]. On the other hand, in sideways crack propagation, relatively less resistance to crack propagation is observed, resulting in fewer chains and entanglements breaking per unit length. The possible reason for sideways crack propagation in Ecoflex 10 can be attributed to the fact that due to higher viscosity, crack tip blunting occurs readily in Shore hardness 10, reducing the stress concentration around the crack markedly. However, in less viscous Ecoflex 30 and 50, there exists a large stress concentration at the crack tip, enabling forward crack propagation.

**Figure 3:**
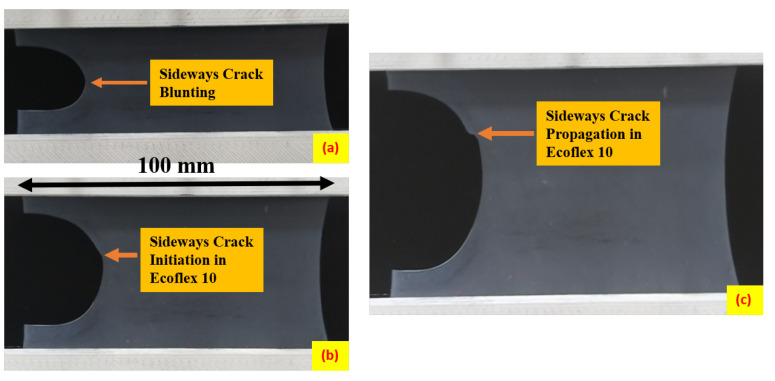
Representation of sideways crack propagation of Ecoflex 10 of thickness 3.6 mm at a strain rate of 40 mm/min; (**a**) blunting of 20 mm sideways crack; (**b**) initiation of sideways crack; (**c**) propagation of sideways crack.

Figure 2(ii) illustrates the sideways crack propagation of Ecoflex 10 with a thickness of 0.6 mm under a pure shear loading at a strain rate of 40 mm/min. The location of the crack without any load applied is depicted in Figure 2(iia), while the initial notch size of 20 mm remains the same during the vertical upward load, as demonstrated in Figure 2(iib). The crack’s blunting does not affect the length of the crack, and after complete blunting, the crack initiates and advances sideways, as presented in Figure 2(iic), avoiding the splitting of the elastomer into two parts. Additionally, Figure 3 provides another instance of sideways crack propagation for a 3.6 mm Ecoflex 10 specimen. The blunting of the sideways crack in the 3.6 mm thick specimen is depicted in Figure 3a, while Figure 3b shows sufficient strain hardening at the tip of the notch after the crack is completely blunted. The chains and entanglements remain strong and unbroken, allowing the crack to propagate sideways, perpendicular to the notch, as demonstrated in Figure 2(iic).

### 2.4. Crack Propagation under Uniaxial Loading

**Figure 4:**
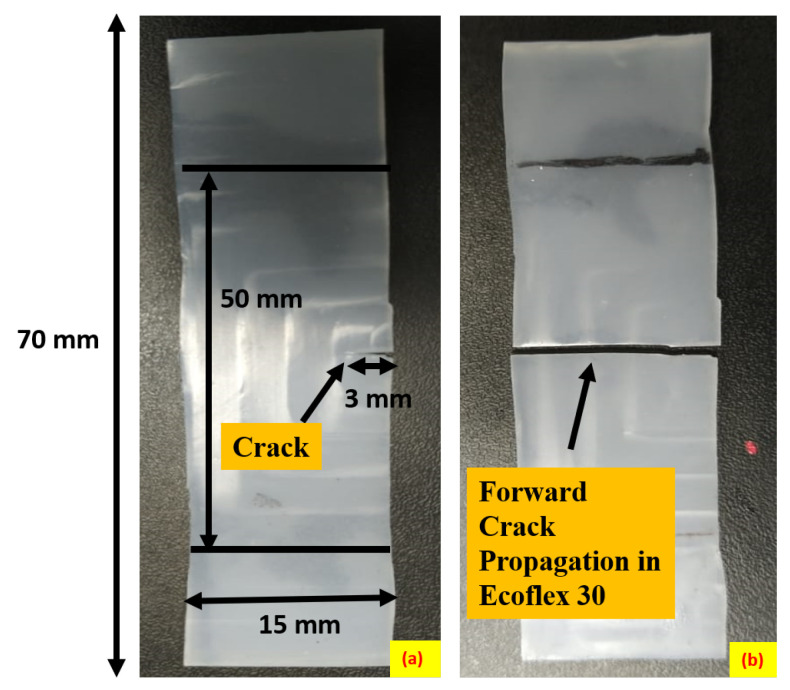
Representation of forward crack propagation of Ecoflex 30 of thickness 3.6 mm at a strain rate of 40 mm/min; (**a**) initial cut in the specimen; (**b**) specimen after forward crack propagation.

**Figure 5:**
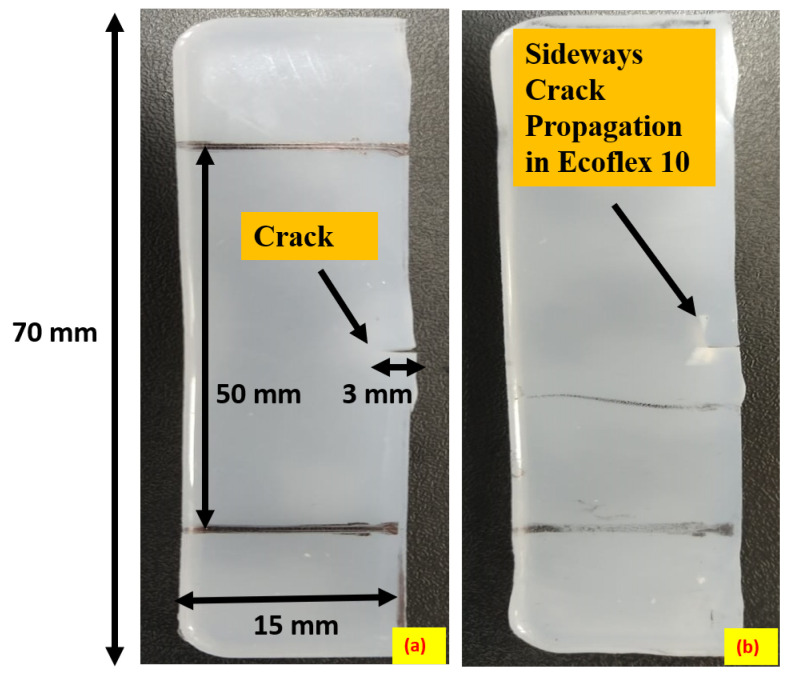
Representation of sideways crack propagation of Ecoflex 10 of thickness 3.6 mm at a strain rate of 40 mm/min; (**a**) initial cut in the specimen; (**b**) specimen after sideways crack propagation.

Figure 4 displays the forward crack propagation of Ecoflex 30 with a thickness of 3.6 mm under uniaxial loading. To investigate the crack propagation behavior of Ecoflex with varying Shore hardness under different deformation modes, a uniaxial tension test is conducted using a UTM. The uniaxial specimen, as illustrated in Figure 4a, is 50 mm in length and 15 mm in width with a 3 mm side notch located in the center of the specimen. Each grip length from the top and bottom of the specimen is approximately 20 mm. As shown in Figure 4b, the crack propagates parallel to the created notch, causing the specimen to fracture into two parts. Additionally, a sideways crack propagation until complete fracture is exhibited in Figure 3c for Ecoflex 10 under uniaxial loading. The sideways crack propagation is observable in Figure 3c as it travels perpendicularly to the cut, reaching the gripper before failing entirely. Furthermore, Figure 5 presents the sideways crack propagation under the uniaxial mode of deformation, as demonstrated in Figure 5a that exhibits the initial specimen with a cut from one side. During uniaxial tensile loading, the specimen elongates in the loading direction, and the notch follows the sideways crack growth, as observed in Figure 5b. This mode also reveals sideways crack propagation.

## 3. Results and Discussion

The results obtained from this research are presented using the two variables of force (N) and displacement (mm). In addition, the strain rates utilized in the tests are presented in millimeters per minute, while the thicknesses of each specimen used in the experiments are reported in millimeters. To ensure accurate and reliable data, each specimen is tested at least three times, and standard deviations are calculated for each case, as can be seen in Table 1. The subsequent section of the paper analyzes various aspects of Ecoflex, including force, fracture toughness, and stretchability, under different Shore hardness, thickness, and strain rates in the context of morphing wing. Additionally, the direction of crack propagation for different Shore hardness is also thoroughly analyzed in the same section, providing a comprehensive overview of the various factors that affect the behavior of Ecoflex.

### 3.1. Effect of Shore Hardness on the Force Versus Displacement Curves and Stretchability at a Fixed Strain Rate

**Figure 6:**
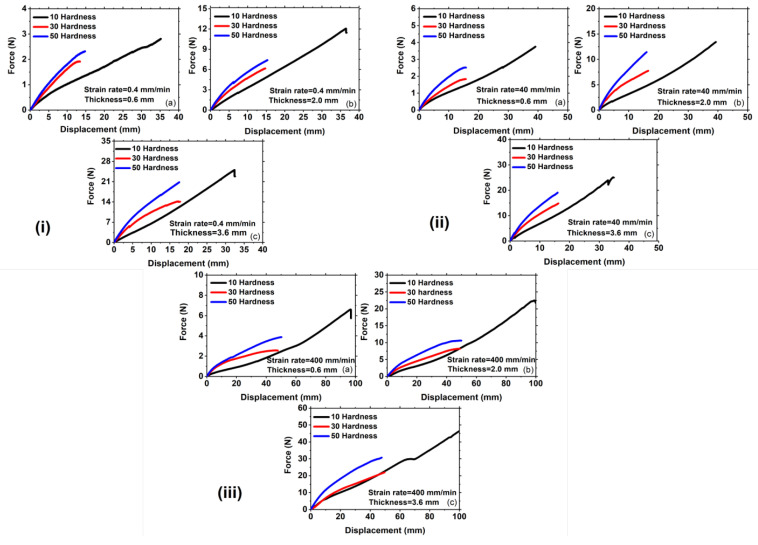
Representation of force versus displacement curves of the specimen under pure shear loading at strain rates of (**i**) 0.4 mm/min for different thicknesses of (**a**) 0.6 mm; (**b**) 2 mm; and (**c**) 3.6 mm, respectively, (**ii**) 40 mm/min for different thicknesses of (**a**) 0.6 mm; (**b**) 2 mm; and (**c**) 3.6 mm, respectively, (**iii**) 400 mm/min for different thicknesses of (**a**) 0.6 mm; (**b**) 2 mm; and (**c**) 3.6 mm, respectively. Note: the scales of the figures are different.

In order to achieve a morphing wing with minimal actuation force during flight, force-displacement curves are plotted for different hardness at three thicknesses in Figure 6. To evaluate the long-term behavior of Ecoflex under monotonic loading, a slow strain rate of 0.4 mm/min is selected, as shown in Figure 6(i). The stretchability of a viscoelastic material is determined by its maximum stretching before failure with a notch. According to previous research [18,20], the stretchability of Ecoflex 30 and 50 is 15 mm, while for Ecoflex 10, it reaches around 40 mm, due to the crack propagating sideways for this Shore hardness, as discussed in detail in Section 2.3 Strain hardening near the crack tip for Ecoflex 10 prevents forward crack propagation, as shown in Figure 2(iib), resulting in the crack turning perpendicular to the cut and advancing sideways. Therefore, Ecoflex 10 does not fail into two parts, but rather the crack advances perpendicular and achieves large stretchability before failure, as shown in Figure 2(iic). This pattern is also observed for other thicknesses, as shown in Figure 6 (iiib,iiic). To investigate the effect of deformation modes on crack propagation, the specimen is tested under uniaxial deformation mode, as shown in Figure 5. Sideways crack propagation is not a function of geometry, but is instead caused by internal chain alignments and structures [22]. The viscosity of Ecoflex 10 is higher than that of Ecoflex 30 and 50, resulting in the chains and entanglements of Ecoflex 10 fully uncoiling for strain hardening at a larger stretch. Fracture toughness ($J_{c}$) of Ecoflex is calculated using the following equation [31,32]:

(1) $$J_{c}=\frac{U}{B(W-a)}$$

where, *U* = area under the force-displacement curve (N-mm);

*B* = thickness of the specimen (mm);

*W* = width of the specimen (mm);

*a* = notch length of the specimen (mm).

During the experiment, the maximum force value can be identified by observing a sudden drop in the force value, which occurs due to catastrophic failure. The data of force value after the drop is omitted, and then the force-displacement graph is plotted to obtain the strain energy density (*U*) by calculating the area under the curve. Table 1 shows that the fracture toughness ($J_{c}$) increases with the increase in Shore hardness for 30 and 50 but is exceptionally high for Ecoflex 10. Figure 6(ii) represents the force-displacement curves at a moderate strain rate of 40 mm/min, where Ecoflex 30 has a stretchability of around 15 mm, whereas it reaches 60 mm for Ecoflex 10 for thicknesses 2 mm and 3.6 mm, as shown in Figure 6(iib,iic), respectively. The stretchability is approximately 60 mm for all thicknesses at a strain rate of 40 mm/min. The force-displacement curves are plotted for different Shore hardness at a fixed strain rate of 400 mm/min for different thicknesses, as shown in Figure 6(iii). It is noted that the force-displacement curves increase from lower (10 H) to higher hardness (30 H) for each thickness, as shown in Figure 6(iiia–iiic), respectively. This is because Ecoflex with higher Shore hardness possesses more stiffness and hysteresis losses, leading to more stretchability than the lower one [18]. In the case of 10 hardness, the crack propagates perpendicular to the notch cut, causing the force-displacement curves to keep increasing until they attain a substantial stretchability of around 100 mm. The crack propagation direction in the case of Ecoflex 10 is not forward but sideways, as the strain hardening near the crack tip occurs quickly, resisting the crack growth in the forward direction [22]. The viscosity of Ecoflex 10 is higher than Ecoflex 30 and 50, as mentioned in the datasheet, which may cause quick strain hardening around the notch tip, leading to sideways crack propagation [22,33].

In contrast, the stretchability of Ecoflex 30 and 50 is approximately 60 mm for all thicknesses, exhibiting a consistent trend. Consequently, the fracture toughness of Ecoflex with a Shore hardness of 10 is consistently higher than that of the higher hardness of 30 and 50. The fracture toughness values are calculated for each thickness and are depicted in Figure 6 (iiia–iiic). At a strain rate of 40 mm/min, the fracture toughness for a 0.6 mm thickness and a hardness of 10 is roughly 4 kJ/m${}^{2}$, whereas for hardness 30 and 50, the fracture toughness values are 0.81 kJ/m${}^{2}$ and 1.20 kJ/m${}^{2}$, respectively, as shown in Table 1. This pattern is consistent across all thicknesses, as depicted in Figure 6(iiib,iiic), with Ecoflex 10 consistently exhibiting higher fracture toughness. This can be attributed to the fact that the crack changes direction and propagates perpendicular to the notch cut in Ecoflex 10. Due to its higher viscosity, more rapid strain hardening takes place around the crack, as previously discussed, which causes the crack to deviate perpendicular to the notch direction. Thus, the force value does not experience a sudden drop for Ecoflex 10, as shown in Figure 6(iii), while the force value drops for Ecoflex 30 and 50 at around 60 mm.

According to the analysis, the stretchability, actuation load, and fracture toughness of Ecoflex are notably influenced by the Shore hardness. Ecoflex 10, in particular, requires less actuation force to elongate the morphing wing. Additionally, the crack propagation in this material occurs sideways, resulting in higher fracture toughness. These properties hold true for all thicknesses of the material.

### 3.2. Effect of Thicknesses on the Force Versus Displacement Curves and Fracture Toughness for Different Shore Hardness at A Fixed Strain Rate

**Figure 7:**
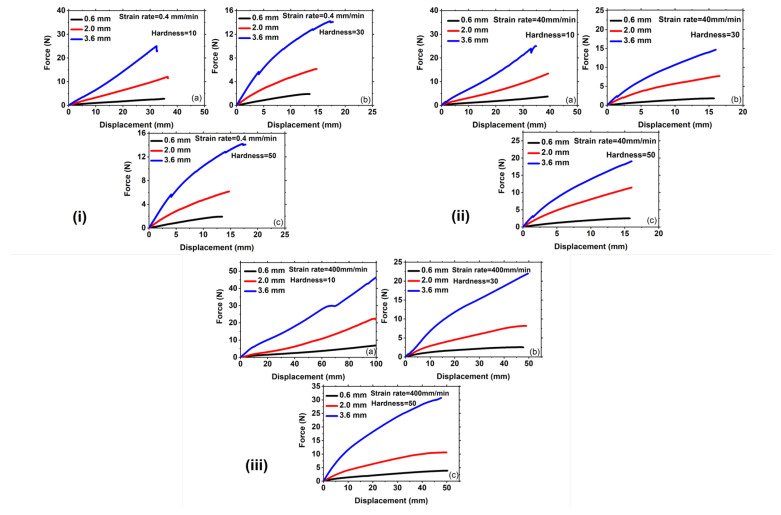
Representation of force versus displacement curves of the specimen under pure shear loading at a strain rate of (**i**) 0.4 mm/min for different hardness of (**a**) 10; (**b**) 30; and (**c**) 50, respectively, (**ii**) 40 mm/min for different thicknesses of (**a**) 10; (**b**) 30; and (**c**) 50, respectively, (**iii**) 400 mm/min for different thicknesses of (**a**) 10; (**b**) 30; and (**c**) 50, respectively. Note: the scales of the figures are different.

**Figure 8:**
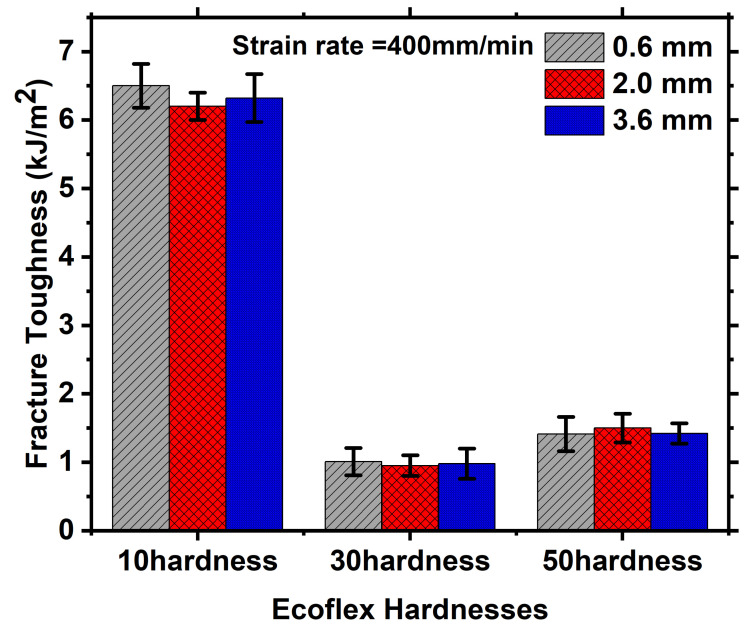
Bar diagram to represent the fracture toughness for different hardness of 0.6 mm, 2 mm and 3.6 mm, respectively.

Figure 7 illustrates the influence of Ecoflex thickness on force-displacement curves for various Shore hardness at a constant strain rate. The analysis compares three different thicknesses, namely 0.6 mm, 2 mm, and 3.6 mm, at a very slow strain rate of 0.4 mm/min as shown in Figure 7(i). The force required to achieve a specific displacement increases with the increase in thickness for Ecoflex 10, as demonstrated in Figure 7(ia). A similar pattern can be observed for other hardness as depicted in Figure 7(ib,ic), respectively. The force necessary for greater thickness is always more because more material volume containing more crosslinks and chains require more actuation load, as shown in Figure 7(i–iii). Furthermore, the stretchability of Ecoflex 10 is higher than that of Ecoflex 30 and 50 for all thicknesses. At a fixed strain rate, the fracture toughnesses calculated from the force-displacement graphs using Equation (1) are almost identical for all thicknesses, as shown in Figure 8 and Table 1. This is due to the fact that fracture toughness is a material property that remains the same for all thicknesses at a given notch.

Additionally, the impact of thickness on force-displacement curves at a moderate strain rate of 40 mm/min is demonstrated in Figure 7(ii), and the same trend is observed as that of a slower strain rate. As depicted in Figure 7(iib), the force at 20 mm strain is 5N, 10N, and 13N for thicknesses of 0.6 mm, 2 mm, and 3.6 mm, respectively, indicating that the force required for a particular strain increases with the thickness of Ecoflex. In addition, the stretchability of Ecoflex 10 is found to be remarkably higher than that of Ecoflex 30 and 50 for all thicknesses, as shown in Figure 7(iia–iic), respectively. For instance, the stretchability of Ecoflex 10 is around 35 mm for all thicknesses, while Ecoflex 30 and 50 exhibit a stretchability of approximately 15 mm, as displayed in Figure 7(iia–iic), respectively. Moreover, Figure 7iii depicts the effect of thickness on the force-displacement curve at a fixed strain rate of 400 mm/min for all Shore hardness. In this case, the force required for a specific displacement is found to be higher for greater thicknesses. This trend is valid for all Shore hardness, as presented in Figure 7(iia–iic), respectively. Consequently, the study of thickness demonstrates that the actuation load demand is lower for thinner thicknesses and higher for thicker thicknesses for all Shore hardness at a specific strain. Additionally, Table 1 shows the fracture toughness with the increase in thickness. For instance, at a strain rate of 400 mm/min and 2 mm thickness, the fracture toughness of Ecoflex 10 is approximately 6.5 kJ/m${}^{2}$ for all thicknesses. On the other hand, Ecoflex 30 and 50 exhibit fracture toughness values of around 1.80 kJ/m${}^{2}$ and 2.51 kJ/m${}^{2}$, respectively, for the same condition. Interestingly, Ecoflex 10 shows sideways crack propagation in both pure shear and uniaxial mode of deformation, as presented in Figure 2(ii) and Figure 5, respectively. In contrast, forward crack propagation is observed for Ecoflex 30 and 50 under the same condition, as depicted in Figure 2(i) and Figure 4 for both pure shear and uniaxial mode of deformation, respectively. Hence, the stretchability and fracture toughness of Shore hardness 30 and 50 are lower than that of Shore hardness 10, as shown in Table 1.

### 3.3. Effect of Strain Rates on the Force Versus Displacement Curves and Fracture Toughness for Different Shore Hardness at A Fixed Thickness

**Figure 9:**
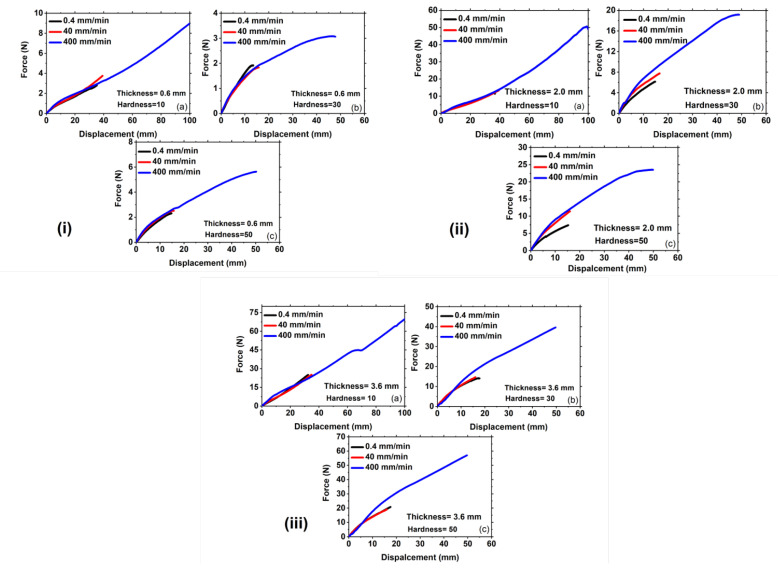
Representation of force versus displacement curves of the specimen under pure shear loading at thicknesses of (**i**) 0.4 mm for different hardness of (**a**) 10; (**b**) 30; and (**c**) 50, respectively, (**ii**) 2.0 mm for different hardness of (**a**) 10; (**b**) 30; and (**c**) 50, respectively, (**iii**) 3.6 mm for different thicknesses of (**a**) 10; (**b**) 30; and (**c**) 50, respectively. Note: the scales of the figures are different.

**Figure 10:**
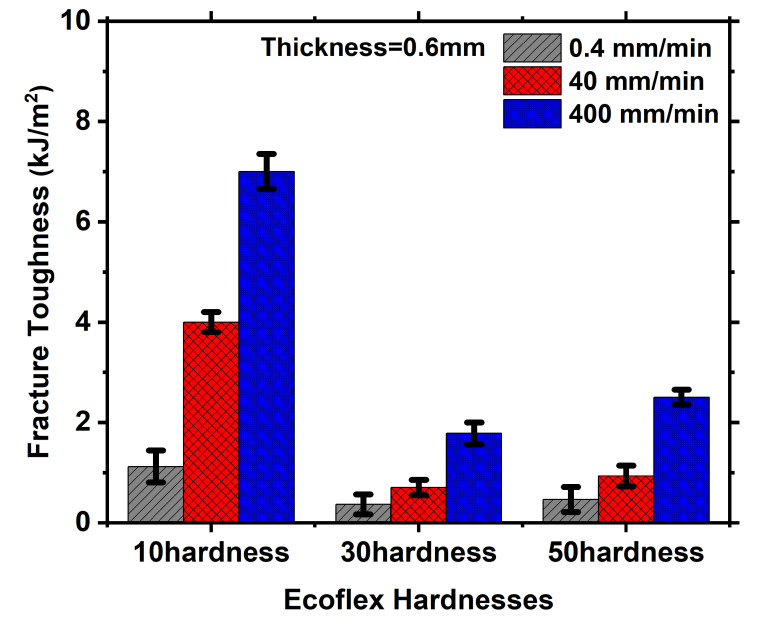
Bar diagram to represent the fracture toughness for different strain rate.

The morphing wing’s deformation rate, or actuation rate, is crucial in practical applications as it varies depending on the situation [14]. Therefore, it is vital to comprehend the Ecoflex skin’s fracture behavior at different strain rates. To test Ecoflex with varying thicknesses at a specific hardness, three strain rates (0.4 mm/min, 40 mm/min, and 400 mm/min) were chosen, as depicted in Figure 9. The force-displacement curves of Ecoflex 10 at a fixed thickness of 0.6 mm were plotted for different strain rates, as shown in Figure 9(ia). It is evident that the force required to achieve a particular displacement increases with the strain rate. This pattern holds true for other hardness values, such as 30 and 50, as shown in Figure 9(ii,iii), respectively. This increase in force occurs because, at higher strain rates, the chains and entanglements have less time to relax, requiring more load to stretch the material. This observation is consistent with previous work by Pharr et al. [34]. The stretchability of Ecoflex slightly increases from 30 to 50 at a particular thickness, as shown in Figure 9(ii,iii). However, the stretchability of Ecoflex 10 is very high, as discussed earlier. The same strain rate effect is observed for a thickness of 2 mm. However, Ecoflex is not as viscous as other elastomeric materials such as Latex, Oppo, and VHB [6,18,34]. When the thickness is 0.6 mm, the stretchability of Ecoflex is slightly increased for 30 and 50 hardness, but it is high for Ecoflex 10. This pattern is consistent for larger thicknesses, such as 2 mm and 3.6 mm, as shown in Figure 9(ii,iii), respectively. Figure 10 and Table 1 clearly show that the fracture toughness increases with increasing strain rates for all thicknesses. For example, at 10 Shore hardness and 2 mm thickness, the fracture toughnesses are 1.10 kJ/m${}^{2}$, 3.98 kJ/m${}^{2}$, and 6.21 kJ/m${}^{2}$ for 0.4 mm/min, 40 mm/min, and 400 mm/min, respectively. Similarly, at 30 Shore hardness, the fracture toughnesses are 0.28 kJ/m${}^{2}$, 0.90 kJ/m${}^{2}$, and 1.81 kJ/m${}^{2}$ for 0.4 mm/min, 40 mm/min, and 400 mm/min, respectively, as shown in Figure 10 and Table 1.

## 4. Future Research Directions

The findings of this study provide valuable insights into the fracture and crack propagation behavior of silicone-based elastomeric skins under multiaxial loading, and opens the door for future research to optimize their design for morphing wing applications. One possible future direction could be to investigate the effect of different types of flaws or notches on the failure properties of elastomeric skins, and develop approaches to minimize their impact. More detailed microstructural and chemical study is also encouraged to understand the unique Sideways crack propagation behavior in the Ecoflex materials. Additionally, the impact of environmental conditions such as temperature, humidity, and UV exposure on the mechanical properties of Ecoflex could be explored to assess its suitability for long-term use in various morphing wing structures. On the basis of current work following the work of King et al. [35,36] and Bishay et al. [37], soft metamaterials with tunable stiffness and poisson’s ratio can be developed adding fibers and controlling their orientation. The new elastomeric composite materials with higher stretngth, improved stretchability and fracture toughness and improved performance can be compared with that of Ecoflex under different loading conditions. Since the study found that the actuation force increases with strain rate, future research could examine the relationship between actuation force and strain rate in more detail, and explore ways to reduce the actuation force required to achieve multiaxial morphing. Furthermore, the use of numerical simulations and modeling techniques could help to predict the crack propagation behavior of elastomeric skins under different loading conditions, and enable the design of more reliable and safe morphing wings. Overall, the findings of this study provide a useful foundation for future research on the design and optimization of elastomeric skins for morphing wing applications.

## 5. Concluding Remarks

Silicone-based elastomeric skins have revolutionized morphing wings with their stretchability, toughness, and low weight. However, flaws or notches can lead to sudden failure, posing a significant challenge for safe operation. Hence, it is crucial to investigate failure properties for reliable design. This study comprehensively analyzes fracture and crack propagation of Ecoflex elastomeric skin. The contributions of this work are as follows:

- Ecoflex 10 with a thinner profile exhibits superior mechanical properties, making it the ideal choice for implementation in morphing wings. With the lowest actuation force requirement compared to other hardness values, it is particularly useful for polymorphing wings, where minimizing actuation load is crucial for both span and camber morphing.
- Ecoflex 30 and 50 show forward crack propagation, while Ecoflex 10 exhibits sideways crack propagation that prevents complete fracture under multiaxial deformation. This property is particularly beneficial in span morphing, where the sideways crack propagation prevents complete failure of the skin.
- Actuation force and fracture toughness of Ecoflex 10, 30, and 50 increase with higher strain rates and Shore hardness, while remaining independent of thickness. At a strain rate of 0.4 mm/min, Ecoflex 10 displays fracture toughness of approximately 1.1 kJ/m${}^{2}$ for all thicknesses. As camber morphing occurs at higher strain rates, Ecoflex’s higher fracture toughness makes it suitable for use in camber morphing.
- Ecoflex 10 exhibits higher stretchability and fracture toughness due to its unique sideways crack propagation when compared to Ecoflex 30 and 50. Ecoflex 10 achieves a stretchability of 60 mm for thicknesses of 2 mm and 3.6 mm, whereas Ecoflex 30 reaches only 15 mm. Therefore, Ecoflex 10 is ideal for span morphing applications that require greater skin stretchability.

## Figures and Tables

**Table 1:** Fracture toughness of Ecoflex at different strain rates and Shore hardness for all three thicknesses under pure shear loading.

Strain Rates/ Shore Hardness		10	30	50	Units
0.4 mm/min	0.6 mm 2 mm 3.6 mm	1.12 1.10 1.15	0.30 0.28 0.35	0.41 0.38 0.36	kJ/m${}^{2}$
40 mm/min	0.6 mm 2 mm 3.6 mm	4.12 3.98 4.05	0.81 0.90 0.89	1.20 1.18 1.14	kJ/m${}^{2}$
400 mm/min	0.6 mm 2 mm 3.6 mm	6.15 6.21 7.12	1.78 1.81 1.78	2.60 2.51 2.71	kJ/m${}^{2}$

## Data Availability

Not applicable.
